# Maternal Dietary Nutrient Intake and Its Association with Preterm Birth: A Case-control Study in Beijing, China

**DOI:** 10.3390/nu9030221

**Published:** 2017-03-01

**Authors:** Yan Zhang, Hong Zhou, Anthony Perkins, Yan Wang, Jing Sun

**Affiliations:** 1 Department of Child, Adolescent and Women’s Health, School of Public Health, Peking University, Beijing 100871, China; zhangyan_sph@bjmu.edu.cn (Y.Z.); hongzhou@bjmu.edu.cn (H.Z.); 2 Menzies Health Institute Queensland, Griffith University, Queensland 4222, Australia; a.perkins@griffith.edu.au; 3 School of Medicine, Griffith University, Queensland 4222, Australia

**Keywords:** maternal dietary intake, nutrients, preterm birth

## Abstract

This study aimed to evaluate dietary nutrient intake among Chinese pregnant women by comparison with Chinese Dietary Reference Intakes (DRIs) and to explore the association between dietary nutrients and preterm birth. A case-control design was conducted in Beijing with 130 preterm delivery mothers in case group and 381 term delivery mothers in control group. Information on mothers’ diet was collected using a food frequency questionnaire, and nutrients and energy intakes were subsequently calculated based on DRIs. Multivariate analysis of variance was used to compare the differences between term and preterm groups in relation to dietary nutrients. Dietary nutrient intakes were imbalanced in both groups compared with Chinese DRIs. Preterm delivery mothers had a lower level of fat and vitamin E intake than term delivery mothers (*p* < 0.05). Multivariate analysis showed lower vitamin E intake in preterm delivery mothers with a prepregnancy BMI < 18.5 kg/m^2^ (*p* < 0.05) and higher carbohydrate intake in preterm delivery mothers with prepregnancy BMI ≥ 24 kg/m^2^ (*p* < 0.05). An imbalanced diet in both groups and low level of dietary intakes of fat and vitamin E in preterm group suggest health education measures should be taken to improve the dietary quality of pregnant women, especially for those with an abnormal prepregnancy BMI.

## 1. Introduction

Maternal nutrition during pregnancy plays an important role in providing the necessary nutrients for fetal growth [1]. An imbalance in maternal nutrition may be a key factor associated with preterm birth [2]. Preterm birth is one of the leading causes of neonatal mortality and morbidity, and has long-term adverse consequences such as poor physical growth [3], deficits in executive function [4], as well as behavioral, attention, and learning problems [5,6]. The incidence of preterm birth is estimated to be 11.1% worldwide, and China has over 1.1 million preterm births per year, which is the second largest number worldwide [7]. Previous studies suggested maternal nutrients maybe associated with low birth weight and preterm birth, such as iron and zinc [8,9]. However, maternal dietary nutrients, as the main source of nutrition for both mothers and fetuses, have been studied less in relation to their association with preterm birth [10,11], especially among Chinese pregnant women. This study aimed to investigate dietary nutrient intake among Chinese pregnant women and explore the association between dietary nutrients and preterm birth using a case-controlled study design in Beijing, China.

## 2. Materials and Methods

### 2.1. Subjects and Design

This was a hospital-based, case-controlled study conducted in Peking University Third Hospital and Haidian Maternal & Child Health Hospital in Beijing, China. These hospitals were chosen due to their high annual maternal delivery data. Pregnant women were recruited from December 2012 to December 2013 when they were admitted for delivery. 

Inclusion criteria for preterm group were giving live birth at less than 37 weeks of gestation without congenital abnormalities or neurological impairments. Matching criteria for the control group were giving live birth at or after 37 and less than 42 weeks of gestation without congenital abnormalities or neurological impairments. A one to three ratio of case-control design was conducted. When one preterm delivery mother was recruited as the case, three term delivery mothers were recruited as controls. The term delivery mothers in the control group were matched to cases within one week of delivery in the same hospital. Exclusion criteria for both groups was giving births to twins, and 60 mothers were excluded. Women who met the inclusion criteria were referred by obstetrical doctors and invited to join in the study. Written informed consents were signed before the interview.

Medical records were reviewed to obtain the age, height, weight, abnormal pregnancy history and pregnancy complications for the included women. Prepregnancy body mass index (BMI) was calculated based on height measured and self-reported prepregnancy weight at the first prenatal clinic visit. BMI was categorized as underweight (<18.5 kg/m^2^), normal weight (18.5–23.9 kg/m^2^), overweight (24.0–27.9 kg/m^2^), and obese (≥28.0 kg/m^2^) based on Criteria of weight for adults in China [12]. In this study, an abnormal pregnancy history was defined as a previous preterm birth, spontaneous abortion or stillbirth. Pregnancy complications mainly included gestational diabetes mellitus (GDM) and hypertensive disorders of pregnancy (HDP). A woman was diagnosed with GDM if she had one or more abnormal values for the 2-h, 75-g oral glucose tolerance test (OGTT) between 24 and 28 weeks of gestation, with the cut-off points of 5.1 mmol/L for fasting, 10.0 mmol/L for 1 hour and 8.5 mmol/L for 2 h [13]. A woman was diagnosed with HDP if her systolic blood pressure (BP) was 140mmHg or greater and/or diastolic BP of 90 mmHg or greater on at least two occasions when tested more than 4 h apart while resting [14].

Maternal interviews were conducted face-to-face by trained medical graduates no later than 3 days after delivery using self-administered questionnaires to collect socio-demographic information (age, nationality, education level, etc.) and life stress during pregnancy. Life stress was defined as negative life experience during pregnancy such as unemployment, death of relatives, and accidents.Study protocols and procedures were reviewed and approved in accordance with the ethical standards of the Medical Ethics Research Board of Peking University (No. IRB00001052-13001).

### 2.2. Dietary Assessment

A validated food frequency questionnaire (FFQ) was used to obtain maternal dietary intake data one month prior to delivery. The FFQ has been previously validated among adults in Beijing [15]. The FFQ included 34 food items which represent most of the common foods in Chinese diet. The food items can be divided into 17 food groups, these were, grains, potatoes, vegetables, fruits, red meats, poultry, animal organs, aquatic products, eggs, dairy products, beans and bean products, nuts, oils, salt, water, beverage, snacks. Details are shown in Table S1. Participants’ food consumption frequencies were recorded for a day, a week or a month. Quantities were assessed using household measurements such as standard sized bowls and teaspoons. A picture book of common foods consumed in China was shown to assist participants. The information provided was added together to calculate participants’ total daily (or weekly) food intake. Next, a food composition database created for this study was used to calculate participants’ nutrient and energy intakes. In the present study, reliability for the FFQ was at good level with Cronbach’s alpha coefficient for internal consistency of 0.66 and Guttman Split-Half Coefficient for Split-Half reliability of 0.84, demonstrating suitability of the FFQ to measure pregnancy women’s dietary intake. Data from the Chinese food composition tables (CFCT) 2004 were used in these calculations [16].

Total energy, five macronutrients and seventeen micronutrients were investigated in total. Nutrient intakes were compared with Recommended Nutrient Intakes (RNIs) or Adequate Intakes (AIs) from Chinese Dietary Reference Intakes (DRIs) 2013 [17].

### 2.3. Statistical Analysis

A Chi-square test was used to compare the characteristics of the preterm and term women participating in this study. Multivariate analysis of variance (MANOVA) was conducted to compare differences in the dietary nutrients of women in preterm and term groups. Confounding factors, including delivery age, abnormal pregnancy history, prepregnancy body mass index (BMI), life stresses and HDP, were controlled in the analysis. Further analyses were conducted according to different prepregnancy BMI groups. Considering the limited number of participants in obese groups, these two groups were merged into one group named as “overweight and obese” group. A *p*-value less than 0.05 was considered significant. Analyses were conducted using SPSS version 23.0 (SPSS Inc., Chicago, IL, USA).

## 3. Results

### 3.1. Descriptive Characteristics of Preterm and Term birth Subjects

A total of 520 mothers from both hospitals (Peking University Third Hospital and Haidian Maternal & Child Health Hospital) were recruited. Among them, 130 mothers had preterm births and 390 mothers had full-term births. A total of 9 mothers in the term group were excluded for incomplete information. Consequently, 381 mothers were included in the term group.

Maternal characteristics are shown in Table 1. The majority of the participants were aged between 25 to 34 years old with a Han nationality. Most women were well educated with a college or above education level and engaged in light physical work during pregnancy. For over 60% of the mothers, the annual family incomes were 40,000 RMB yuan or more per person. Mothers of preterm infants were significantly more likely to have a higher prepregnancy BMI, life stress and HDP during pregnancy compared to mothers of term infants. Differences based on the other health factors (abnormal pregnancy histories and GDM) were not statistically significant.

### 3.2. Energy and Nutrient Intake Compared with DRIs

Energy and nutrient intakes of both groups were compared with Chinese DRIs (See Table 2). The average total energy intake of preterm delivery mothers was below the recommendation (9410 KJ/day for light physical work, 10,670 KJ/day for moderate physical work, and 11,920 KJ/day for hard physical work) while the intake of term delivery mothers was adequate. According to the comparable macronutrient DRIs, both groups had insufficient intakes of protein and fiber. Compared with micronutrient DRIs, about half of the micronutrient intakes were sufficient, but some intake problems were also obvious. For both preterm and term groups, the mean intakes of vitamin A, calcium and iron were much lower; intakes of thiamin, riboflavin and magnesium were slightly lower. Inversely, the mean intake of sodium was much higher, which is equivalent to almost three times the DRI.

### 3.3. Energy and Nutrient Intake between Preterm and Term Groups 

Energy and nutrient intakes between preterm and term groups were compared in Table 2. Women who had preterm births had lower total energy and fat intakes than women who had term births. Preterm delivery mothers had higher intakes of retinol, but lower intakes of niacin, vitamin E, copper and magnesium than term delivery mothers. After adjusting for delivery age, abnormal pregnant history, prepregnancy BMI, life stress and HDP, the difference among fat and vitamin E intake remained significant.

### 3.4. Multivariate Analyses According to Prepregnancy BMI Groups

Table 3 shows the association between dietary nutrient intake and preterm birth according to prepregnancy BMI groups. In the underweight group, total energy and vitamin E intakes tended to be lower in preterm delivery mothers in the raw model, and vitamin E intake was still significantly lower in the preterm group when confounders including delivery age, abnormal pregnant history, life stress, and HDP were adjusted for. In the normal weight group, only niacin tended to be lower in preterm delivery mothers in the raw model. However, there were no significant differences between the two birth groups for all items after confounders were adjusted for. In the overweight and obese group, carbohydrate intake was higher in preterm delivery mothers, and the result was still significant after the confounders were adjusted for. In the overweight and obese group, fat and vitamin E intake tended to be lower in preterm delivery mothers, but the results were not statistically significant after the confounders were adjusted for.

## 4. Discussion

Results showed that both preterm and term groups had imbalanced dietary intakes compared to Chinese DRIs. A comparison of dietary nutrient intakes between the preterm and term groups showed that fat and vitamin E intakes appeared to be associated with preterm births.

### 4.1. Nutrient Intake Compared with DRIs

The mean total energy intake of the preterm group was lower than recent reports in both urban and rural China [18,19]. As most of the participants in our study were well-educated citizens and had a preferable family income, the insufficient energy intake in the preterm group may not be due to limited food resource, but may be associated with less weight gain during pregnancy and smaller infants, which may carry a lower risk of delivery complication [1]. Insufficient protein and fiber intakes were found in both preterm and term groups. The result was similar to a previous study among well-educated and socioeconomically unrestricted Germany pregnant women. These women were thought to have positive factors for a better diet, but poor dietary habits with less nutrient-dense food played a major role [20]. Similar results were also found in China, and the mean reason may be improper dietary habits, for example, limited milk and plant foods, which are good sources of protein and dietary fiber [18,21,22].

Dietary intakes of micronutrients were imbalanced according to the results. The intakes of vitamin A, calcium and iron were much lower than DRIs, which was quite different from white pregnant women in the U.S who were reported to have a higher nutrient-dense diet, but similar to black women, who tended to have energy-dense but nutrient-poor diets [23]. This problem was also found in previous studies in China, and one reason was the lack of healthy diet knowledge, another reason was that good food resources of these nutrients (such as dairy products, red meats and animal organs) were not chosen by pregnant women according to traditional eating habits [18]. Nevertheless, the dietary quality seemed to be improved in some ways, as zinc intake was sufficient compared with a previous report in developing countries [24]. On the other hand, excessive sodium intake was still a big problem as reported before, and this might reflect a common public health problem in China [25].

### 4.2. Macronutrient Intake and Preterm Birth

The results showed that women in the preterm group had lower fat intake than women in the term group. This result is consistent with that found in a previous study in Poland [26]. A proper intake of dietary fat is important in preventing preterm births. The function of fat differs according to the different types of fatty acid components. Short and middle chain fatty acids are mainly used to provide sufficient energy for pregnancy and prevent poor fetal growth and preterm birth [27]. Long-chain polyunsaturated fatty acids (LCPUFAs) affect numerous metabolic and structural processes. For example, *n*-3 LCPUFAs affect the synthesis of bioactive molecules such as prostaglandins, which in turn affect an array of biological activities, including vasodilation, placental blood flow, cervical ripening and the onset of labour [28]. Overall, unsaturated fatty acids play a more important role than saturated fatty acids in preventing preterm births [29]. In the present study, only total fat intake data were obtained; thus, future research is needed to confirm the association between different fatty acids and preterm births.

### 4.3. Micronutrient Intake and Preterm Birth 

Our result suggests sufficient dietary vitamin E may be necessary to prevent preterm birth. A similar result was found by Koenig et al among a small sample of African American women [30]. In the present study, the amount of vitamin E intake in the preterm group was even lower than in rural China [19]. This may reflect a diet preference that preterm delivery mothers dislike foods such as vegetable oils, nuts, fat of meat, cereals and leafy green vegetables [31]. The biological function of vitamin E is an antioxidant that protects tissues and cells from the damaging effect of reactive oxygen species [32]. It may prevent preterm birth by improving the health of the cervix and inhibiting premature cervical remodeling and provide beneficial effect for placenta function [30,33]. 

### 4.4. Association between Dietary Nutrient Intake and Preterm Birth According to Prepregnancy BMI

The present study demonstrated improper dietary intake in both prepregnancy underweight and overweight & obese groups was associated with preterm births. These results are consistent with findings in previous studies [34,35]. A previous study found mothers with low prepregnancy BMI tended to have small gestational age babies and preterm births, though the biological mechanisms were unclear [36]. In our study, preterm delivery mothers in the prepregnancy underweight group showed insufficient nutrient intake, this may be a possible reason of preterm births. A high prepregnancy BMI may increase the risk of obstetric complications and large gestational-age babies, and poor diet quality such as excessive energy intake was considered to worsen the condition [34]. A previous study suggested that obese women should have a lower carbohydrate intake at moderate levels in late gestation for better offspring health [37]. However, in the present study, preterm delivery mothers with a high prepregnancy BMI still showed a higher intake of carbohydrate, which may increase the risk of preterm births.

### 4.5. Strengths and Limitations of the Study

To our knowledge, few studies have focused on dietary nutrient intakes among Chinese pregnant women, especially those who had preterm births. The present study provides information on dietary nutrient intakes among both preterm and term delivery mothers in Beijing, China, and showed the insufficient and excessive intakes of nutrients by comparison with Chinese DRIs, which provides the most recent view of the nutritional status of urban pregnant women in China. Our study also supplemented the evidence from Chinese citizens for the association between dietary nutrients and preterm births, which has not yet been clarified worldwide.

There are a number of limitations of the present study. Firstly, the result of our study may only reflect the condition of pregnant women who had low social-economic risk factors in China, as the study population was mostly well-educated women with a favourable family income. Secondly, the case-controlled study design cannot draw conclusions on causal relationships between dietary nutrients and preterm births. Thirdly, we only captured the dietary intake information in the last month before delivery, which may not exactly represent the dietary intakes of the entire pregnancy period. However, previous studies showed the dietary characteristics and dietary nutrient intakes did not change much through different pregnant periods [18,38]. Finally, we could not subdivide the intakes of different kinds of fatty acids according to the Chinese Food Composition Tables, which did not allow us to able to explain the association between fat and preterm births.

### 4.6. Implications of Research and Practice 

Large-scale studies of women with different social-economical characteristics are needed to provide a comprehensive view of the dietary nutrient intake status of pregnant Chinese women. Additionally, a prospective cohort study design is needed to clarify the association between nutrient intake and preterm births. In practice, health education is needed to provide pregnant women with detailed information on how to appropriately arrange their diet. Further, women’s dietary habits may vary according to prepregnancy BMI; thus, individual advice may be necessary.

## 5. Conclusions

In conclusion, our study results showed dietary nutrient intakes were imbalanced in both preterm and term groups compared with Chinese DRIs, and we demonstrated that women who had preterm births had lower dietary intake of fat and vitamin E than women with full-term births. It is necessary to take measures to improve the dietary quality of pregnant women, especially for those with an abnormal prepregnancy BMI, to optimize their birth outcomes.

## Figures and Tables

**Table 1:** Comparison of maternal characteristics between preterm and term groups.

Variables	Preterm (*n* = 130)	Term (*n* = 381)	χ^2^	*p*
	n (%)	n (%)		
**Demographic Factors**				
Delivery age(years)				
21–24	6 (4.6)	13 (3.4)	2.149	0.542
25–29	53 (40.8)	159 (41.7)	-	-
30–34	51 (39.2)	166 (43.6)	-	-
35–46	20 (15.4)	43 (11.3)	-	-
Nationality				
Han	126 (96.9)	361 (94.8)	1.022	0.312
Minority	4 (3.1)	20 (5.2)	-	-
Education				
Middle school or below	19 (16.1)	50 (13.1)	3.305	0.192
College	78 (66.1)	233 (61.2)	-	-
Graduate or above	21 (17.8)	98 (25.7)	-	-
Occupation				
No work	35 (31.0)	88 (23.0)	3.310	0.191
Light physical work	76 (67.3)	282 (73.8)	-	-
Moderate physical work	2 (1.8)	12 (3.1)	-	-
Annual family income (RMB yuan per person)				
<40,000	31 (31.3)	69 (23.3)	3.553	0.169
40,000–100,000	58 (58.6)	204 (68.9)	-	-
>100,000	10 (10.1)	23 (7.8)	-	-
**Health Factors**				
Abnormal pregnancy history				
Yes	14 (10.8)	53 (13.9)	0.840	0.359
No	116 (89.2)	328 (86.1)	-	-
Prepregnancy BMI				
<18.5	7 (5.7)	50 (13.2)	11.467	0.009
18.5–23.9	78 (63.4)	257 (68.0)	-	-
24–27.9	29 (23.6)	57 (15.1)	-	-
≥28	9 (7.3)	14 (3.7)	-	-
Life stress				
Yes	43 (33.3)	76 (19.9)	9.653	0.002
No	86 (66.7)	305 (80.1)	-	-
HDP				
Yes	47 (36.4)	30 (7.9)	61.323	<0.001
No	82 (63.6)	351 (92.1)	-	-
GDM				
Yes	32 (24.8)	121 (31.8)	2.218	0.136
No	97 (75.2)	260 (68.2)	-	-

Categorical variables are presented as sum and percentages, *p*-values for categorical variables using chi-square test, *p*<0.05 was considered statistically significant. Abbreviation: BMI, body mass index; HDP, hypertensive disorders of pregnancy; GDM, gestational diabetes mellitus.

**Table 2:** Association between nutrient intake and preterm birth.

Energy & Nutrients Intake	Preterm (*n* = 130) M (SD)	Term (*n* = 381) M (SD)	RNI/AI	*p*	*p*’
Total Energy (KJ)	9038.13 (2211.17)	9509.02 (2322.70)	-	0.04	0.36
Macro nutrients					
Carbohydrate (g)	242.52 (74.00)	241.47 (66.83)	-	0.88	0.50
Fat (g)	82.33 (25.17)	92.72 (30.47)	-	<0.01	0.02
Protein (g)	77.61 (19.85)	82.17 (24.65)	85	0.06	0.38
Cholesterol (mg)	557.31 (284.47)	516.47 (171.40)	-	0.06	0.10
Fiber (g)	12.22 (4.28)	13.05 (5.21)	25	0.10	0.41
Micro nutrients					
Vitamin A (μg)	498.70 (222.59)	497.28 (202.47)	770	0.95	0.82
Retinol (μg)	257.09 (119.69)	238.68 (79.40)	-	0.04	0.08
Thiamin (mg)	0.88 (0.36)	0.93 (0.39)	1.5	0.15	0.42
Riboflavin (mg)	1.30 (0.50)	1.33 (0.49)	1.5	0.50	0.84
Niacin (mg NE)	13.99 (4.35)	15.64 (6.00)	12	<0.01	0.09
Vitamin C (mg)	117.30 (60.35)	127.13 (68.17)	115	0.15	0.34
Total Vitamin E (mg)	29.60 (9.51)	33.57 (11.30)	-	<0.01	0.02
Calcium (mg)	743.95 (335.22)	775.46 (332.84)	1000	0.35	0.67
Copper (mg)	1.73 (0.40)	1.87 (0.55)	0.9	0.01	0.19
Iron (mg)	19.19 (6.17)	20.38 (7.00)	29	0.09	0.34
Magnesium (mg)	323.08 (80.91)	351.25 (109.03)	370	<0.01	0.14
Manganese (mg)	5.90 (1.42)	6.11 (1.69)	4.9	0.21	0.82
Phosphorus (mg)	1242.31 (335.73)	1301.79 (378.76)	720	0.11	0.50
Potassium (mg)	2457.93 (736.43)	2595.26 (848.15)	2000	0.10	0.41
Selenium (μg)	91.30 (70.57)	93.68 (66.16)	65	0.73	0.94
Sodium (mg)	4382.67 (1243.94)	4514.12 (1797.13)	1500	0.44	0.45
Zinc (mg)	11.43 (3.40)	12.16 (3.96)	9.5	0.06	0.32

Continuous variables are presented as the mean (standard deviation, SD); multivariate analysis of variance (MANOVA) was used to compare nutrient intakes between the two groups, *p* and *p*’ represent the *p* value before and after adjusting for confounders, respectively; confounders for adjustment include delivery age, abnormal pregnancy histories, prepregnancy body mass index (BMI), life stress, and hypertensive disorders of pregnancy (HDP). RNI: Recommended nutrient intake; AI: Adequate intake.

**Table 3:** Association between dietary nutrient intake and preterm birth according to prepregnancy BMI.

Energy & Nutrient Intake	Underweight				Normal Weight				Overweight & Obese			
	Preterm (*n* = 7) M (SD)	Term (*n* = 50) M (SD)	*p*	*p*’	Preterm (*n* = 78) M (SD)	Term (*n* = 257) M (SD)	*p*	*p’*	Preterm (*n* = 38) M (SD)	Term (*n* = 71) M (SD)	*p*	*p*’
Total Energy (KJ)	8222.34 (822.13)	10051.12 (2380.51)	0.05	0.06	9177.14 (2326.99)	9535.04 (2357.95)	0.24	0.30	9135.63 (2118.65)	8987.51 (2066.76)	0.72	0.33
**Macro nutrients**												
Carbohydrate (g)	211.01 (40.48)	251.20 (65.40)	0.12	0.14	243.21 (75.72)	243.22 (69.09)	1.00	0.89	252.82 (77.84)	226.74 (58.06)	0.05	0.03
Fat (g)	78.52 (13.69)	99.35 (34.99)	0.13	0.15	85.91 (27.83)	91.91 (28.78)	0.11	0.19	78.49 (20.86)	90.60 (32.64)	0.04	0.22
Protein (g)	75.89 (14.19)	88.02 (23.01)	0.18	0.14	78.03 (19.37)	82.98 (25.65)	0.12	0.18	78.62 (20.58)	74.97 (20.82)	0.38	0.17
Cholesterol (mg)	659.17 (435.00)	512.97 (153.85)	0.08	0.16	562.32 (291.24)	518.91 (178.26)	0.11	0.36	535.40 (254.90)	509.68 (162.96)	0.52	0.26
Fiber (g)	10.16 (2.20)	13.02 (4.47)	0.10	0.19	12.54 (4.63)	13.19 (5.50)	0.35	0.40	12.37 (3.85)	12.43 (4.66)	0.95	0.85
**Micro nutrients**												
Vitamin A (μg)	532.40 (150.08)	490.54 (186.96)	0.57	0.87	501.35 (224.57)	505.11 (218.15)	0.90	0.74	495.76 (221.17)	471.27 (150.86)	0.50	0.44
Retinol (μg)	310.01 (177.29)	251.75 (87.67)	0.16	0.35	262.82 (123.09)	236.30 (79.88)	0.03	0.14	239.14 (105.41)	238.40 (72.34)	0.97	0.52
Thiamin (mg)	0.86 (0.14)	1.00 (0.38)	0.34	0.24	0.87 (0.34)	0.95 (0.41)	0.10	0.13	0.91 (0.40)	0.82 (0.27)	0.16	0.12
Riboflavin (mg)	1.39 (0.25)	1.46 (0.59)	0.76	0.45	1.31 (0.50)	1.34 (0.49)	0.64	0.55	1.27 (0.50)	1.22 (0.34)	0.51	0.32
Niacin (mg NE)	13.16 (2.72)	17.03 (6.12)	0.11	0.15	14.10 (4.28)	15.78 (6.07)	0.02	0.06	14.35 (4.81)	14.09 (5.51)	0.81	0.58
Vitamin C (mg)	101.31 (24.80)	118.58 (51.80)	0.39	0.44	120.24 (63.33)	130.06 (73.22)	0.29	0.32	118.56 (59.55)	121.06 (59.41)	0.84	0.95
Total Vitamin E (mg)	24.71 (4.63)	34.87 (11.91)	0.03	0.04	30.90 (10.16)	33.59 (11.33)	0.06	0.17	28.44 (7.86)	32.42 (10.80)	0.04	0.22
Calcium (mg)	791.30 (100.23)	867.91 (427.24)	0.64	0.33	749.80 (333.32)	778.09 (336.21)	0.52	0.54	723.39 (344.55)	703.06 (216.20)	0.71	0.53
Copper (mg)	1.52 (0.21)	1.95 (0.59)	0.06	0.11	1.76 (0.40)	1.88 (0.56)	0.10	0.22	1.75 (0.40)	1.76 (0.49)	0.91	0.78
Iron (mg)	17.72 (2.30)	21.10 (6.31)	0.17	0.11	19.14 (5.88)	20.81 (7.56)	0.07	0.12	19.72 (6.52)	18.28 (4.85)	0.19	0.12
Magnesium (mg)	287.54 (37.44)	372.82 (117.24)	0.06	0.09	328.71 (81.05)	353.24 (111.17)	0.07	0.16	326.23 (81.26)	327.18 (92.33)	0.96	0.77
Manganese (mg)	5.21 (1.53)	6.23 (1.55)	0.11	0.20	5.93 (1.33)	6.12 (1.80)	0.38	0.64	6.19 (1.50)	5.94 (1.37)	0.40	0.39
Phosphorus (mg)	1228.09 (155.27)	1413.35 (415.67)	0.25	0.16	1253.36 (338.68)	1308.20 (385.87)	0.26	0.30	1243.35 (340.41)	1198.49 (301.40)	0.48	0.25
Potassium (mg)	2327.87 (313.04)	2694.85 (811.15)	0.24	0.22	2517.44 (785.62)	2609.75 (875.44)	0.40	0.42	2437.87 (680.94)	2458.81 (772.61)	0.89	0.79
Selenium (μg)	97.78 (31.94)	106.03 (70.91)	0.76	0.46	86.69 (60.72)	96.64 (71.35)	0.27	0.33	95.52 (83.76)	74.57 (33.27)	0.07	0.09
Sodium (mg)	4349.24 (615.85)	4439.07 (1269.23)	0.86	0.83	4451.44 (1308.18)	4460.44 (1625.42)	0.96	0.95	4200.32 (1204.97)	4741.52 (2558.14)	0.22	0.43
Zinc (mg)	11.22 (1.82)	13.04 (3.77)	0.22	0.15	11.42 (3.25)	12.31 (4.15)	0.08	0.12	11.70 (3.74)	11.01 (3.15)	0.31	0.19

Continuous variables are presented as the mean (standarddeviation, SD); multivariate analysis of variance (MANOVA) was used to compare nutrient intakes between the two groups; *p* and *p*’ represent the *p* value before and after adjusting for confounders, respectively; confounders for adjustment including delivery age, abnormal pregnancy histories, life stress, and hypertensive disorders of pregnancy (HDP).

## Supplementary Materials

The following are available online at http://www.mdpi.com/2072-6643/9/3/221/s1, Table S1: Food groups and items used in the food frequency questionnaire (FFQ).
