# Supplementary material for: Maternal Dietary Nutrient Intake and Its Association with Preterm Birth: A Case-control Study in Beijing, China
Source: Nutrients. 2017 Mar 1;9(3):221. doi: 10.3390/nu9030221 (PMC5372884; doi:10.3390/nu9030221)
Supplement: Supplementary file 1 [file nutrients-09-00221-s001.docx]

Supplementary Materials

**Table S1.** Food groups and items used in food frequency questionnaire(FFQ)

| **Food groups** | **Food items** |
| --- | --- |
| Grains | Rice, flour, other grains |
| Potatoes | Potato/sweet potato/taro |
| Vegetables | Dark vegetable, light vegetable, fungus |
| Fruits | Apple/pear/banana/strawberry |
| Red meats | Pork, beef /mutton |
| Poultry | Chicken/duck/goose |
| Animal organs | Animal liver, animal blood |
| Aquatic products | Fish/prawn |
| Eggs | Eggs |
| Dairy products | Fresh milk, yogurt, milk powder, cheese |
| Beans and bean products | Soybean/other beans, bean products |
| Nuts | Peanut, sunflower |
| Oils | Oils |
| Salt | Salt |
| Water | Water |
| Beverage | Fresh fruit juice, commercial fruit vegetable juice, milk drink, carbonated drinks |
| Snacks | Cookie pastries, fruit vegetable products, puffed food, confectionery, fast food, meat snack |
